# Prognosis of “pre-heart failure” clinical phenotypes

**DOI:** 10.1371/journal.pone.0231254

**Published:** 2020-04-10

**Authors:** Raghava S. Velagaleti, Meghan I. Short, Martin G. Larson, Ramachandran S. Vasan

**Affiliations:** 1 Framingham Heart Study, Framingham, Massachusetts, United States of America; 2 Department of Medicine, Cardiology Section, Boston VA Healthcare System, West Roxbury, Massachusetts, United States of America; 3 Department of Biostatistics, Boston University School of Public Health, Boston, Massachusetts, United States of America; 4 Department of Medicine, Preventive Medicine and Cardiology Sections, School of Medicine, and Department of Epidemiology, School of Public Health, Boston University, Boston, Massachusetts, United States of America; Azienda Ospedaliero Universitaria Careggi, ITALY

## Abstract

**Background:**

Heart failure (HF) is a clinical syndrome where diagnostic certainty varies. The prognosis of individuals with some clinical features of HF, but without the fully overt syndrome, is unclear. Therefore, we sought to evaluate their natural history.

**Methods and results:**

Between 1990 and 2009, all suspected HF cases in the Framingham Heart Study were adjudicated into 3 groups reflecting varying diagnostic certainty: definite (meeting HF diagnostic criteria; n = 479), possible (meeting HF criteria but with an alternative explanation for findings; n = 135), and probable (insufficient criteria for definite HF; n = 121) HF. Age-and-sex-matched individuals (n = 1112) without HF or cardiovascular disease (CVD) were controls. Using multivariable-adjusted Cox regression, we compared the possible/probable HF groups with controls regarding risk of incident definite HF, coronary heart disease (CHD), other CVD or death; and with definite HF regarding risk of latter three outcomes.

During follow-up (mean 8.6 years), ~90% of individuals with possible, probable and definite HF experienced CVD events or died. Compared with controls, those with possible or probable HF experienced higher hazards for definite HF, CHD, other CVD and death (hazards ratios [HR] 1.35–9.31; p<0.05). The possible/probable groups did not differ from the definite HF group for risk of any outcome. Compared with the possible HF group, the probable HF group had a higher propensity for definite HF (HR 1.64, with a higher proportion of ischemic HF) but lower risk of death (HR 0.69).

**Conclusions:**

Individuals meeting partial criteria for HF are at a substantial risk for progression to HF, CVD, and mortality.

## Introduction

Heart failure (HF) is a complex syndrome without any pathognomonic clinical features. [1] Laboratory tests aid the diagnosis of HF in certain settings, [2] and echocardiographic evaluation can help define its etiological and pathophysiological subsets. Nonetheless, the individual clinical features of HF suffer from overall poor sensitivity and specificity. [3, 4] Therefore, a syndromic approach to HF diagnosis combines several key symptoms and signs with laboratory and/or imaging findings serving as corroborating/supporting evidence. [5] In epidemiological studies, a diagnosis of HF is established by applying a well-validated set of criteria, [6] in the absence of a competing explanation for the clinical findings. These criteria typically combine symptoms and signs of systemic and pulmonary venous congestion with pathophysiological responses (S3 gallop or tachycardia) weighted into ‘major’ and ‘minor’ criteria based on their sensitivities and specificities.

**Fig 1:**
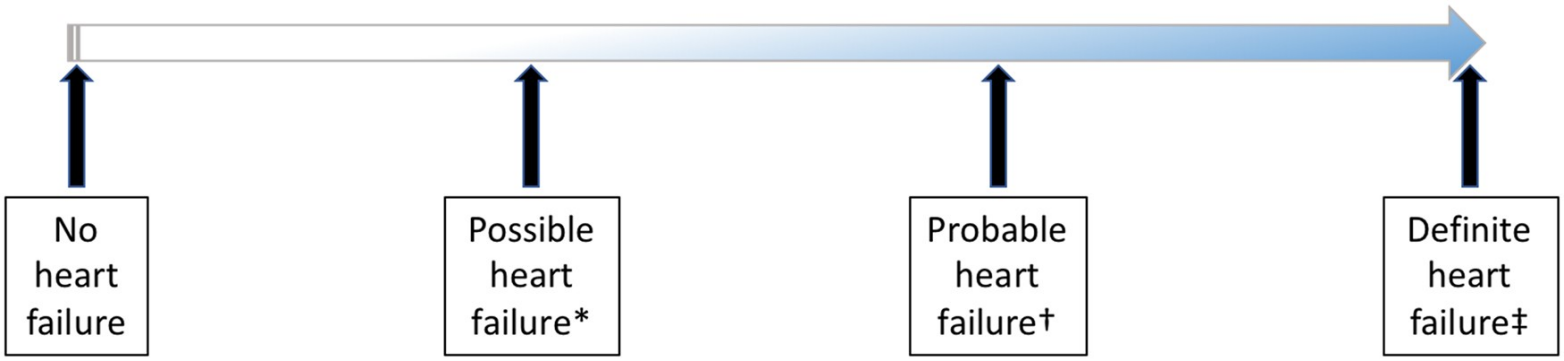
Clinical uncertainty in establishing heart failure diagnosis.

In epidemiological settings, when suspected HF events are reviewed by an adjudication panel, participants are categorized according to a range of diagnostic certainties, from possible, probable to definite HF (Fig 1). For example, at the Framingham Heart Study (FHS), participants who have some clinical features suggestive of HF, but do not meet the full criteria (2 major or 1 major plus 2 minor criteria required for definite HF; see S1 Appendix) are categorized as “probable HF”. Similarly, participants who meet criteria for definite HF but have an alternate explanation for some of the clinical findings are labeled as “possible HF”. The clinical course of these two groups of participants who meet partial criteria or full criteria but with other coexisting conditions is unclear. It is conceivable that such participants are simply at an earlier stage along the HF continuum, i.e. they are in a “pre-HF” state. As such, they can potentially serve as ways to identify individuals at high risk for progression to overt HF and/or incidence of other major cardiovascular (CVD) events. It is noteworthy that the identification of at-risk individuals based on risk factors, screening biomarkers or echocardiography is challenged by the low positive predictive value of these tests, i.e., only a small proportion of individuals with abnormal screening tests go on to develop HF. The natural history of pre-HF phenotypes has not been described previously.

In this investigation, we sought to evaluate (a) if participants with the presumed “pre-HF” states differ from both normal controls and those with definite HF with respect to subsequent incidence of CVD events and death; and (b) if they differ from each other in terms of distinct patterns of mortality, incident HF and/or subsequent cardiovascular events.

## Methods

### Study sample configuration

The FHS Original (n = 5209) and Offspring (n = 5124) cohorts have been described. [7] Participants without HF before 1990 and evaluated between 1/1/1990 and 12/31/2009 constituted the present sampling frame. A total of 735 participants were reviewed for HF, classified into one of three groups reflecting varying degrees of diagnostic certainty, and included in the current study. An additional 1112 participants constituted the control (referent) group described below. The follow-up period was until 12/31/2015. All participants provided written informed consent, and the investigation was approved by the Institutional Review Board of Boston University and complies with the Declaration of Helsinki.

### Case definition

All participants with features suspicious for HF were reviewed by a panel of three experienced physicians. The adjudication process included scrutiny of outpatient medical records, hospitalization records and FHS research visit charts (the FHS makes a sustained effort to obtain all medical records of participants from their physicians and hospitals they were treated at). Participants who fulfilled the FHS criteria for HF [8] (listed in S1 Appendix) were categorized as definite HF. Those who had some clinical criteria but fell short of a diagnosis of definite HF (e.g., with one major criterion, or 2 minor criteria, or 1 major criterion plus one minor criterion) were termed “probable HF”. Participants who met HF criteria but had an alternative explanation for findings (such as lung, kidney or liver disorders) were termed “possible HF”. The classification was based on the findings at the first instance a given participant was reviewed. If a participant was diagnosed with both possible and probable HF during separate reviews at different timepoints, the earliest of those diagnoses was used. Of note, the diagnoses of possible, probable and definite HF were based on the application of Framingham criteria and clinical judgement of the panel of reviewers and did not incorporate information from ancillary testing.

### Controls

Individuals who attended FHS research visits contemporaneous with participants in above groups and without any prevalent CVD or any HF criteria at baseline, were eligible. Each participant with possible or probable HF was matched with up to 6 participants who were eligible controls at the same baseline exam when the diagnosis is made, on the basis of sex and 5-year age strata (mean of 4.3 matched controls per case). Because it was possible for a control to contribute multiple eligible baseline exams, a control could be matched with multiple cases at different baseline exams. When this occurred, a case was chosen at random for each control, so that no controls were sampled multiple times. The resulting sample of controls consisted of n = 1112 participants.

### Baseline characteristics and covariates definitions

Baseline characteristics and covariate information were drawn from the FHS “baseline examination” for each participant which was chosen to be the nearest FHS examination within four years prior or two years following the diagnosis of possible, probable, or definite HF.

Body mass index was calculated as weight (in kilograms) divided by height (in meters) squared. Systolic blood pressure was the average of two physician obtained readings on the left arm of seated participants at the FHS research visit. Hypertension treatment was coded as “yes” if the participant received any anti-hypertensive medication in the year preceding the baseline examination and was otherwise coded as “no”. Diabetes was defined as current or prior diagnosis of diabetes, or fasting glucose ≥ 126 mg/dl, or non-fasting glucose ≥ 200 mg/dl, as measured at baseline examination. Current smoking was defined as cigarette smoking at the time of, or one-year preceding, the baseline examination. Total cholesterol and high-density lipoprotein cholesterol were measured utilizing standard laboratory methods. Estimated glomerular filtration rate (eGFR) was calculated using the CKD-Epi formula. [9] Prevalent coronary heart disease (CHD) included recognized myocardial infarction, acute coronary syndrome and angina pectoris. Prevalent lung disease included a clinical diagnosis of chronic bronchitis, emphysema, chronic obstructive pulmonary disease and asthma.

### Outcome definition

Incident definite HF, non-fatal CHD, non-fatal other CVD, and all-cause mortality were the four mutually exclusive endpoints of interest for comparisons between the probable HF vs. possible HF vs. control group; the latter three also served as endpoints for comparisons between probable HF vs. possible HF vs. definite HF. Incident CHD included recognized myocardial infarction, acute coronary syndrome and angina pectoris. Other CVD included ischemic and hemorrhagic stroke, transient ischemic attack, and intermittent claudication. For exploratory analyses, we also categorized HF as follows. Based on echocardiographic ejection fraction (EF) close to the time of incident HF, we termed HF as HFrEF if EF was < 50% and HFpEF if EF ≥ 50%. In addition, incident definite HF events were termed “ischemic” HF if they were preceded by either myocardial infarction or coronary insufficiency; otherwise, they were deemed “non-ischemic” HF. All events were reviewed and adjudicated by a panel of three physicians using published criteria. [10–12]

### Statistical analyses

The following analyses tested whether probable and possible HF groups differ from either the control group or definite HF. In all four groups, we calculated incident event proportions for outcomes of interest. We compared possible and probable HF groups with the control group for risk of definite HF, CHD, other CVD and death, using Cox proportional hazards regression models [13] stratified by 5-year age groups and sex, and adjusting for age, then additionally adjusting for body mass index, systolic blood pressure, hypertension treatment, current smoking and prevalent CHD. Next, to evaluate whether possible and probable HF differ from definite HF regarding subsequent risk of CHD, other CVD and death, we fitted Cox models with definite HF as the referent group. Methods for determining whether the proportional hazards assumption was met and addressing cases where it is violated are provided in the **Supplementary Information Section 2**.

To assess whether possible and probable HF differ from each other, we performed the following analyses. We constructed the multivariable Cox models as described above with two groups: probable HF versus possible HF (referent); the outcomes were definite HF, CHD, other CVD and death. In exploratory analyses, to assess whether possible and probable HF are differentially prone to subsequent incidence of HFrEF and HFpEF, in each group we calculated (a) event proportions of incident HFpEF and HFrEF in both groups and (b) event proportions of ischemic and non-ischemic HF. And lastly, in a subset of participants in whom b-type natriuretic peptide (BNP) measurements at the time of the initial pre-HF or definite HF event were available, we calculated median and interquartile range of this biomarker in the possible, probable and definite HF groups. We performed a Kruskal-Wallis test to assess for overall difference between the three groups and Wilcoxon tests to assess between-group pairwise comparisons.

All statistical analyses were performed with SAS software version 9.4 (Cary, NC). We used a two-sided significance level, alpha = 0.05, for each statistical test.

## Results

We present baseline characteristics of study participants in Table 1. All groups comprised elderly individuals (mean age ≥ 75 years) and over half were women. In the HF groups, over two-thirds were treated for hypertension and a third had diabetes. In all groups, mean eGFR was borderline normal. Probable and definite HF groups had a higher proportion of CHD, whereas the possible HF had higher proportions of smokers and prevalent lung disease.

**Table 1:** Baseline characteristics of study participants.

Variables	Controls N = 1112	Possible HF* N = 135	Probable HF† N = 121	Definite HF N = 479
**Age, years**	75 ± 11	77 ± 11	78 ± 11	79 ± 11
**Women, (%)**	647 (58)	83 (61)	66 (55)	240 (50)
**Body Mass Index, kg/m**^**2**^	26.5 ± 4.6	27.6 ± 6.9	28.4 ± 5.4	28.1 ± 5.5
**Systolic Blood Pressure, mmHg**	137 ± 20	140 ± 25	140 ± 24	143 ± 24
**Hypertension Treatment, (%)**	502 (45)	75 (56)	79 (66)	323 (69)
**Current or Previous Diabetes, (%)**	118 (13)	30 (31)	25 (29)	135 (37)
**Smoking, (%)**	89 (8)	23 (17)	14 (12)	40 (8)
**Total cholesterol/HDL cholesterol ratio**	4.1 ± 1.4	4.1 ± 1.7	4.3 ± 1.7	4.4 ± 1.8
**Estimated GFR, ml/min/1.73m**^**2**^	68 ± 19	65 ± 19	63 ± 23	59 ± 22
**Prevalent CHD**‡, **(%)**	84 (8)	34 (25)	48 (40)	247 (52)
**Prevalent Lung Disease**§, **(%)**	145 (16)	51 (44)	37 (36)	108 (27)

Cells present mean ± SD for continuous variables and n (%) for categorical variables.

* Meet HF criteria but have an alternative explanation for findings.

^†^ Do not meet full criteria for definite HF.

^‡^ Includes recognized myocardial infarction, coronary insufficiency and angina pectoris.

^§^Includes chronic obstructive pulmonary disease, chronic bronchitis, emphysema and asthma.

HF = heart failure; HDL = high density lipoprotein; GFR = glomerular filtration rate; CHD = coronary heart disease.

The various groups were followed up for mean 8.6 (maximum 28.3 years). Table 2 and S1 Table display proportions of incident events and causes of death during follow-up respectively. The proportion who developed definite HF was 3-fold higher in the probable HF group compared with controls, and 2-fold compared with the possible HF group. The proportions of CHD death were several-fold higher in the groups with probable and definite HF compared with possible HF or controls. In contrast, probable or definite HF had lower proportions of cancer death and other death compared with possible HF.

**Table 2:** Proportions of incident events on follow-up in the four study groups.

Event	Controls N = 1112	Possible HF* N = 135	Probable HF† N = 121	Definite HF N = 479
**Definite HF**	162 (15)	28 (21)	51 (42)	—
**CHD**‡	135 (12)	20 (15)	16 (13)	77 (16)
**Other CVD**§	190 (17)	18 (13)	19 (16)	66 (14)
**All-cause Mortality**	697 (63)	122 (90)	106 (88)	429 (90)

Cells present observed frequencies—n (%)—of incident events in each group. Of note, column percentages do not add up to 100% as participants could have incurred 1 or more non-fatal events prior to death and all events are shown in the counts.

* Meet HF criteria but have an alternative explanation for findings.

^†^ Do not meet full criteria for definite HF.

^‡^ Includes recognized myocardial infarction, coronary insufficiency and angina pectoris.

^§^ Includes ischemic stroke, hemorrhagic stroke, transient ischemic attack and intermittent claudication.

HF = heart failure; CHD = coronary heart disease; CVD = cardiovascular disease.

**Fig 2:**
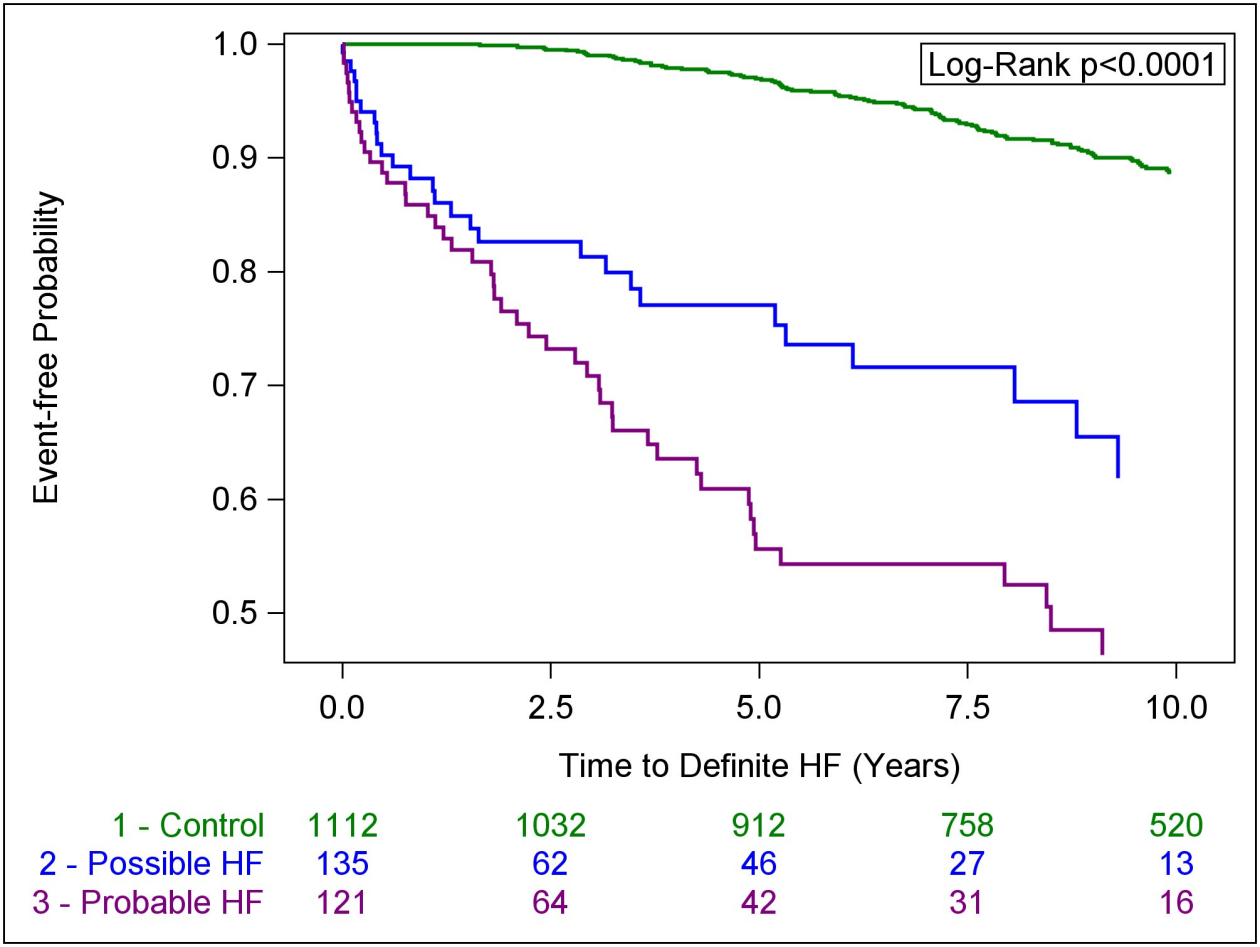
Kaplan-Meier curves for incident definite HF in control, possible HF and probable HF groups.

Results from Cox regression models comparing possible and probable HF groups with control group are shown in Table 3. For these comparisons, the assumption of proportionality of hazards was violated; compared with controls, we noted a several fold higher risk of all outcomes in the possible and probable HF groups (Table 3 and Fig 2), but there was variation of hazards with time (higher hazards earlier during follow-up and attenuation later; details in S1 Data and S2, S3 and S4 Tables). However, even at 5 years of follow-up, the possible and probable HF groups incurred three-to-six-fold higher hazards for definite HF and death, compared with controls (S3 and S4 Tables). For comparisons of possible and probable HF groups with definite HF group, the assumption of proportionality of hazards was confirmed. Neither possible HF nor probable HF carried hazards for CHD, other CVD or death that were statistically significantly different from the definite HF group (Table 4).

**Table 3:** Risk of various clinical events in those with possible and probable HF compared with controls.

	Controls	Possible HF*	Probable HF†	p-value‡
**A. Definite HF**				
**Event Proportion, no. of events /no. at risk, (%)** §	162/1112 (15)	28/135 (21)	51/121 (42)	**N/A**
**Age-and-sex-adjusted Hazards Ratio (CI)**	**1.00 REFERENT**	6.21 (3.74–10.32)	9.31 (6.17–14.05)	<0.0001
**Multivariable-adjusted Hazards Ratio (CI)** ||	**1.00 REFERENT**	4.74 (2.74–8.21)	7.10 (4.52–11.13)	<0.0001
**B. CHD**				
**Event Proportion, no. of events /no. at risk, (%)** §	135/1112 (12)	20/135 (15)	16/121 (13)	**N/A**
**Age-and-sex-adjusted Hazards Ratio (CI)**	**1.00 REFERENT**	3.79 (2.19–6.56)	1.86 (1.07–3.23)	<0.0001
**Multivariable-adjusted Hazards Ratio (CI)** ||	**1.00 REFERENT**	3.06 (1.66–5.64)	1.35 (0.71–2.56)	0.0039
**C. Other CVD**				
**Event Proportion, no. of events /no. at risk, (%)** §	190/1112 (17)	18/135 (13)	19/121 (16)	**N/A**
**Age-and-sex-adjusted Hazards Ratio (CI)**	**1.00 REFERENT**	1.94 (1.11–3.39)	1.78 (1.07–2.97)	0.0019
**Multivariable-adjusted Hazards Ratio (CI)** ||	**1.00 REFERENT**	1.85 (1.02–3.33)	1.61 (0.93–2.76)	0.0126
**D. Death**				
**Event Proportion, no. of events /no. at risk, (%)** §	697/1112 (63)	122/135 (90)	106/121 (88)	**N/A**
**Age-and-sex-adjusted Hazards Ratio (CI)**	**1.00 REFERENT**	5.17 (4.04–6.60)	3.34 (2.64–4.24)	<0.0001
**Multivariable-adjusted Hazards Ratio (CI)** ||	**1.00 REFERENT**	5.14 (3.92–6.75)	3.38 (2.58–4.43)	<0.0001

* Meet HF criteria but have an alternative explanation for findings.

^†^ Do not meet full criteria for definite HF.

^‡^ p-value for whether either pre-HF category as a whole predicts time to the outcome

^§^ Event proportions are based on overall sample.

^||^ Model adjusted for age, sex, SBP, antihypertensive medications, current smoking, prevalent CHD, and body mass index.

HF = heart failure; CHD = coronary heart disease; CVD = cardiovascular disease.

**Table 4:** Risk of various events in those with possible and probable HF, compared with definite HF.

	Definite HF	Possible HF*	Probable HF†	p-value‡
**A. CHD**				
**Event Proportion, no. of events /no. at risk, (%)** §	77/479 (16)	20/135 (15)	16/121 (13)	
**Age-and-sex-adjusted Hazards Ratio (CI)**	**1.00 REFERENT**	0.95 (0.58–1.56)	0.69 (0.41–1.19)	0.31
**Multivariable-adjusted Hazards Ratio (CI)** ||	**1.00 REFERENT**	0.98 (0.57–1.67)	0.63 (0.35–1.12)	0.26
**B. Other CVD**				
**Event Proportion, no. of events /no. at risk, (%)** §	66/479 (14)	18/135 (13)	19/121 (16)	**N/A**
**Age-and-sex-adjusted Hazards Ratio (CI)**	**1.00 REFERENT**	0.95 (0.56–1.60)	0.96 (0.58–1.61)	0.82
**Multivariable-adjusted Hazards Ratio (CI)** ||	**1.00 REFERENT**	1.01 (0.58–1.76)	1.03 (0.61–1.75)	0.92
**C. Death**				
**Event Proportion, no. of events /no. at risk, (%)** §	429/479 (90)	122/135 (90)	106/121 (88)	**N/A**
**Age-and-sex-adjusted Hazards Ratio (CI)**	**1.00 REFERENT**	1.13 (0.93–1.39)	0.84 (0.68–1.04)	0.74
**Multivariable-adjusted Hazards Ratio (CI)** ||	**1.00 REFERENT**	1.21 (0.97–1.51)	0.83 (0.66–1.05)	0.97

* Meet HF criteria but have an alternative explanation for findings.

^†^ Do not meet full criteria for definite HF.

^‡^ p-value for whether either pre-HF category as a whole predicts time to the outcome.

^§^ Event proportions are based on overall sample.

^||^ Model adjusted for age, sex, SBP, antihypertensive medications, current smoking, prevalent CHD, and body mass index.

HF = heart failure; CHD = coronary heart disease; CVD = cardiovascular disease.

Comparisons between possible and probable HF showed that probable HF had a higher risk of progression to definite HF, whereas possible HF had a higher risk for all-cause mortality (S5 Table). The groups did not differ significantly with respect to CHD and other CVD risk. In exploratory analyses, of the probable and possible HF groups who went on to develop definite HF, 46 (90%) and 27 (96%) respectively had EF information available at the time of incident definite HF diagnosis. The proportions of HFrEF and HFpEF were 11/27 (41%) and 16/27 (59%) in the possible HF group and 19/46 (41%) and 27/46 (59%) in the probable HF group. Likewise, the proportions of ischemic HF and non-ischemic HF were 6/28 (21%) and 22/28 (79%) in the possible HF group and 22/51 (43%) and 29/51 (57%) in the probable HF group. And lastly, BNP measurements at the time of the initial event were available in a subset of possible (18/135; 13%), probable (15/121; 12%) and definite HF (81/479; 17%) participants. The median (quartile 1, quartile 3) values in these groups were 396 pg/ml (170, 699), 558 pg/ml (447, 874) and 839 pg/ml (560, 1420) respectively, and were statistically significantly different from each other (p<0.0001); i.e. median BNP values significantly increased from possible to probable to definite HF groups. The differences between groups was also significant in pairwise comparisons (p-values: p = 0.039 for possible HF vs probable HF; p = 0.043 for probable HF vs. definite HF; p<0.0001 for possible HF vs. definite HF).

## Discussion

### Principal findings

In our community-based cohorts of individuals, we noted that possible and probable HF (serving as “pre-HF” phenotypes) were associated with a substantial incidence of cardiovascular events and mortality compared to age-and-sex-matched controls. Accounting for coexisting comorbidities attenuated these relations only modestly. Only for the comparisons of probable and possible HF groups with the control group, we noted variation of hazards over time such that markedly higher hazards for definite HF were noted initially that attenuated over time. However, it is noteworthy that the risk for definite HF, CHD and death were statistically significantly higher in the possible and probable HF groups, compared with the control group, even at five years of follow-up. Of note, the risk of CVD and death in these participants is comparable with that experienced by individuals with definite HF, a group that is known to carry a high morbidity and mortality burden. [14] Although some key differences emerged between possible and probable HF groups in terms of types of disease burden (cardiac vs. non-cardiac disease burden and causes of death), either designation connoted poor prognosis.

### Context of our findings and comparisons to prior literature

There is a paucity of literature (and no widely agreed upon consensus) on what constitutes a pre-HF phenotype. The American Heart Association/American College of Cardiology framework for the staging of HF has 2 pre-clinical stages: Stage A (those with HF risk factors) and Stage B (those with asymptomatic abnormalities in left ventricular structure and/or function). Risks of progression to HF in those with Stage A [15] or Stage B [16, 17] have been reported. There has been considerable interest in further refining HF risk prediction in those without overt HF, with many studies addressing individual biomarkers [18, 19] or consolidated multimarker risk scores [20] for prediction of incident HF. These approaches to identifying people prior to onset of HF, although demonstrating high relative risk, are typically limited by low absolute risk (thus low positive predictive value), therefore constituting sub-optimal screening strategies. Although some evidence exists for biomarker-guided management approaches to preventing HF, it comes with almost doubling of additional testing [21] and uncertain cost effectiveness.

In our current investigation, we utilized a clinical framework of manifested abnormalities that do not reach the threshold for a diagnosis of HF, to identify 2 groups of individuals who can be deemed to be in a pre-HF state and assessed their future risk for progression to definite HF and occurrence of other major CVD events of clinical interest. It is conceivable that either the designations we used or a “clinical decision rule” based on a parsimonious set of clinical features comprising HF criteria can serve as ways to identify those at a high absolute risk for progression to future HF, non-fatal CVD and CV mortality (almost 90% of our elderly participants in the possible and probable HF groups had some major event or death during follow-up). Alternately, such clinical grouping can facilitate implementing biomarker testing (such as BNP) more effectively to identify individuals at highest risk of developing overt HF especially in resource poor settings, a premise that warrants further study.

### Alternative explanations for our findings

Several alternate explanations for the findings in our investigation need to be considered. It is possible that these participants are manifesting a “forme fruste” of HF. Alternately, it is conceivable that some in possible and probable groups may be misdiagnosed and not be classified as true HF category owing to variable temporal expression of clinical features. Another explanation is that the criteria used for diagnosis of HF also serve as markers for multisystem impairments. Indeed, FHS investigators have previously reported that subclinical abnormalities in several non-cardiac organ systems predict incident HF [22] and the various criteria that the pre-HF groups were positive for are merely indicators for these abnormalities.

Lastly, there may be misclassification based on use of FHS criteria alone in the adjudication of HF. Our framework did not use blood-based (e.g. BNP) or imaging (e.g. EF) biomarkers in the diagnosis of HF; it is conceivable that in clinical settings, the use of such information may have led a proportion of participants with probable HF to be characterized as definite HF, despite a paucity of clinical features. Similarly, with BNP levels and EF information in hand, the alternative explanation for findings could be discounted and definite HF diagnosis potentially assigned in those with possible HF.

### Strengths and limitations

Our investigation is strengthened by prospective collection of all data, careful phenotyping of case groups by a physician panel, and consistent assessment of events via uniform application of the same criteria over time. However, several limitations need to be acknowledged. We did not have adequate statistical power to fully elucidate if the possible and probable HF groups had differential propensity for HFpEF versus HFrEF. Temporal trends in comorbidity burden and in types of HF may limit the applicability of the findings to the current era. We did not have information regarding diastolic function parameters to further characterize those participants with HFpEF. The lack of BNP information in all participants is a limitation, especially in participants with lung disease, kidney disease etc. in whom symptoms similar to HF may occur and BNP aids HF diagnosis. It is indeed important to note the possibility that our categories of probable and possible HF may reflect incomplete diagnosis, and not a true pre-clinical stage of HF. We did consider restricting our study sample to a more contemporary era when such information was routinely available; however, this would have made it difficult to have adequate follow-up time to accrue clinical events in follow-up and gain statistical power. As information regarding cardiovascular events that occurred in the community could only be obtained from clinical medical records, misclassification owing to lack of data is possible. A modest sample size limited our ability to further characterize the groups in terms of specific types of CVD outcomes. Lastly, all participants were elderly white individuals of European ancestry and findings may not be generalizable to other age and/or ethnic groups.

## Conclusions

In a community-based sample we noted that individuals with clinical features that connote increased likelihood of (but not diagnostic for) HF carry a substantial burden of morbidity and mortality. Although they differ somewhat with respect to their propensity for progression to definite HF and cause-specific mortality, a categorization of either possible or probable HF carries a dismal prognosis. Given the higher propensity for progression to definite HF (compared to normal individuals) and substantial subsequent CVD burden, they may potentially serve as “pre-HF” phenotypes. Whether use of these classifications serves the purpose of screening for those at high risk for progression to HF or whether targeted management strategies in them improves future outcomes warrants further investigation.

## Supporting information

S1 Table Causes of death in the 4 study groups. Methods for assessing the proportionality of hazards and their interpretation.
(DOCX)

S2 Table Tests for significance of interactions between log(time) and possible HF or probable HF status. (DOCX)

S3 Table Age-and-sex-adjusted analyses comparing possible and probable HF to controls, accounting for variation of hazards over time. (DOCX)

S4 Table Multivariable-adjusted analyses comparing possible and probable HF to controls, accounting for variation of hazards over time. (DOCX)

S5 Table Comparisons between possble and probable HF. (DOCX)

S1 Appendix Framingham Heart Study criteria for HF diagnosis. (DOCX)

S1 Data (DOCX)
