# Supplementary material for: Prognosis of “pre-heart failure” clinical phenotypes
Source: PLoS One. 2020 Apr 10;15(4):e0231254. doi: 10.1371/journal.pone.0231254 (PMC7147998; doi:10.1371/journal.pone.0231254)
Supplement: S1 Table — Methods for assessing the proportionality of hazards and their interpretation. (DOCX) [file pone.0231254.s001.docx]

**Supplementary Table 1: Causes of death in the four study groups.**

| **Death from** | **Controls**  **N = 697** | **Possible HF***  **N = 122** | **Probable HF†**  **N = 106** | **Definite HF**  **N = 429** | **P for differences** |
| --- | --- | --- | --- | --- | --- |
| **CHD**‡ | 53 (8) | 4 (3) | 14 (13) | 96 (22) | <0.0001 |
| **Other CVD§** | 120 (17) | 19 (16) | 25 (24) | 117 (27) | 0.0003 |
| **Cancer** | 117 (17) | 20 (16) | 11 (10) | 48 (11) | 0.036 |
| **Other cause**\|\| | 329 (47) | 69 (57) | 45 (42) | 135 (31) | <0.0001 |
| **Unknown** | 78 (11) | 10 (8) | 11 (10) | 33 (8) | 0.25 |

Note: “N”s in the heading row indicate total number of events in each group during the follow-up period. Each cell shows numbers of events within study group for each category and column percentage.

“P-value” in last column derived from unadjusted chi-square tests for differences in proportions across row.

* Meet HF criteria but have an alternative explanation for findings.

† Do not meet full criteria for definite HF.

‡ Includes recognized myocardial infarction, coronary insufficiency and angina pectoris.

**§** Includes ischemic stroke, hemorrhagic stroke, transient ischemic attack and intermittent claudication.

|| Death from all other causes that are not CVD or cancer related.

HF = heart failure; CHD = coronary heart disease; CVD = cardiovascular disease.
