# Supplementary material for: Prognosis of “pre-heart failure” clinical phenotypes
Source: PLoS One. 2020 Apr 10;15(4):e0231254. doi: 10.1371/journal.pone.0231254 (PMC7147998; doi:10.1371/journal.pone.0231254)
Supplement: S2 Table — (DOCX) [file pone.0231254.s002.docx]

**Supplementary Table 2. Tests for significance of interactions between log(time) and possible HF or probable HF status.**

| **Outcome** | **Adjusted for** | **p-value for PH Violation for Possible HF vs Control** | **p-value for PH Violation for Probable HF vs Control** |
| --- | --- | --- | --- |
| **Definite HF** | Age, sex | <0.0001*** | <0.0001*** |
|  | Age, sex, MV | 0.0003*** | <0.0001*** |
| **CHD** | Age, sex | 0.0019** | 0.0265* |
|  | Age, sex, MV | 0.0081** | 0.0059** |
| **Other CVD** | Age, sex | 0.17 | 0.06 |
|  | Age, sex, MV | 0.0015** | 0.50 |
| **Death** | Age, sex | <0.0001*** | 0.0009*** |
|  | Age, sex, MV | <0.0001*** | <0.0001*** |

*p<0.05; **p<0.01; ***p<0.001. Lower p-value indicates more significant violation of the PH assumption.

PH = proportional hazards; HF = heart failure; CHD = coronary heart disease; CVD = cardiovascular disease; MV = multivariable.
