# Supplementary material for: Prognosis of “pre-heart failure” clinical phenotypes
Source: PLoS One. 2020 Apr 10;15(4):e0231254. doi: 10.1371/journal.pone.0231254 (PMC7147998; doi:10.1371/journal.pone.0231254)
Supplement: S3 Table — (DOCX) [file pone.0231254.s003.docx]

**Supplementary Table 3. Age-and-sex-adjusted analyses comparing possible and probable HF to controls, accounting for variation of hazards over time.**

|  | **Controls** | **Possible HF*** | **Probable HF†** | **p-value‡** |
| --- | --- | --- | --- | --- |
| 1. **Definite HF** | | | | |
| **1 year post-baseline**  **Hazards Ratio (CI)** | **1.00**  **REFERENT** | 62.3  (19.5-199.1) | 94.1  (30.2-293.4) | <0.0001 |
| **5 years post-baseline**  **Hazards Ratio (CI)** | **1.00**  **REFERENT** | 3.12  (1.54-6.29) | 6.76  (4.09-11.15) | <0.0001 |
| 1. **CHD** | | | | |
| **1 year post-baseline**  **Hazards Ratio (CI)** | **1.00**  **REFERENT** | 4.71  (2.58-8.59) | 2.32  (1.29-4.18) | <0.0001 |
| **5 years post-baseline**  **Hazards Ratio (CI)** | **1.00**  **REFERENT** | 1.86  (0.87-4.00) | 1.25  (0.61-2.58) | 0.14 |
| 1. **Death** | | | | |
| **1 year post-baseline**  **Hazards Ratio (CI)** | **1.00**  **REFERENT** | 7.23  (5.42-9.63) | 4.74  (3.50-6.41) | <0.0001 |
| **5 years post-baseline**  **Hazards Ratio (CI)** | **1.00**  **REFERENT** | 3.63  (2.71-4.88) | 2.99  (2.31-3.87) | <0.0001 |

* Meet HF criteria but have an alternate explanation for findings.

† Do not meet full criteria for definite HF.

‡ p-value for whether belonging to either pre-HF category predicts time to the outcome. Model includes an interaction term for either pre-HF category with time.

HF = heart failure; CHD = coronary heart disease; CVD = cardiovascular disease.
