# Supplementary material for: Prognosis of “pre-heart failure” clinical phenotypes
Source: PLoS One. 2020 Apr 10;15(4):e0231254. doi: 10.1371/journal.pone.0231254 (PMC7147998; doi:10.1371/journal.pone.0231254)
Supplement: S4 Table — (DOCX) [file pone.0231254.s004.docx]

**Supplementary Table 4. Multivariable-adjusted analyses comparing possible and probable HF to controls, accounting for variation of hazards over time.**

|  | **Controls** | **Possible HF*** | **Probable HF†** | **p-value‡** |
| --- | --- | --- | --- | --- |
| 1. **Definite HF** | | | | |
| **1 year post-baseline**  **Hazards Ratio (CI)** | **1.00**  **REFERENT** | 18.96  (7.86-45.74) | 43.89  (16.40-117.4) | <0.0001 |
| **5 years post-baseline**  **Hazards Ratio (CI)** | **1.00**  **REFERENT** | 5.44  (3.03-9.76) | 9.69  (5.84-16.05) | <0.0001 |
| 1. **CHD** | | | | |
| **1 year post-baseline**  **Hazards Ratio (CI)** | **1.00**  **REFERENT** | 8.70  (3.39-22.34) | 6.52  (1.93-22.03) | <0.0001 |
| **5 years post-baseline**  **Hazards Ratio (CI)** | **1.00**  **REFERENT** | 2.98  (1.57-5.66) | 1.79  (0.93-3.48) | 0.0459 |
| 1. **Other CVD** | | | | |
| **1 year post-baseline**  **Hazards Ratio (CI)** | **1.00**  **REFERENT** | 10.31  (3.23-32.98) | PH assumption met | 0.0057 |
| **5 years post-baseline**  **Hazards Ratio (CI)** | **1.00**  **REFERENT** | 2.20  (1.17-4.15) | PH assumption met | 0.016 |
| 1. **Death** | | | | |
| **1 year post-baseline**  **Hazards Ratio (CI)** | **1.00**  **REFERENT** | 8.61  (6.07-12.21) | 6.08  (4.16-8.87) | <0.0001 |
| **5 years post-baseline**  **Hazards Ratio (CI)** | **1.00**  **REFERENT** | 3.50  (2.54-4.83) | 3.09  (2.32-4.11) | <0.0001 |

Hazards ratios are from models that adjusted for age, sex, systolic blood pressure, antihypertensive medications, current smoking, prevalent CHD, and body mass index in the groups compared to referent.

* Meet HF criteria but have an alternate explanation for findings.

† Do not meet full criteria for definite HF.

‡ p-value for whether belonging to either pre-HF category predicts time to the outcome. Model includes an interaction term for either pre-HF category with time.

HF = heart failure; CHD = coronary heart disease; CVD = cardiovascular disease.
