# Supplementary material for: Prognosis of “pre-heart failure” clinical phenotypes
Source: PLoS One. 2020 Apr 10;15(4):e0231254. doi: 10.1371/journal.pone.0231254 (PMC7147998; doi:10.1371/journal.pone.0231254)
Supplement: S5 Table — (DOCX) [file pone.0231254.s005.docx]

**Supplementary Table 5. Comparison between possible and probable HF.**

|  | **Possible HF*** | **Probable HF†** | **p-value** |
| --- | --- | --- | --- |
| 1. **Definite HF** | | | |
| **Event Proportion, no. of events / no. at risk, (%)** | 25/124 (20) | 45/107 (42) | **N/A** |
| **Hazards Ratio (CI) ‡** | **1.00**  **REFERENT** | 1.64  (0.99-2.72) | 0.054 |
| 1. **CHD** | | | |
| **Event Proportion, no. of events / no. at risk, (%)** | 18/124 (15) | 14/107 (13) | **N/A** |
| **Hazards Ratio (CI) ‡** | **1.00**  **REFERENT** | 0.60  (0.29-1.25) | 0.17 |
| 1. **Other CVD** | | | |
| **Event Proportion, no. of events / no. at risk, (%)** | 18/124 (15) | 18/107 (17) | **N/A** |
| **Hazards Ratio (CI) ‡** | **1.00**  **REFERENT** | 0.92  (0.47-1.84) | 0.82 |
| 1. **Death** | | | |
| **Event Proportion, no. of events / no. at risk, (%)** | 111/124 (90) | 93/107 (87) | **N/A** |
| **Hazards Ratio (CI) ‡** | **1.00**  **REFERENT** | 0.69  (0.52-0.93) | 0.013 |

* Meet HF criteria but have an alternative explanation for findings.

† Do not meet full criteria for definite HF.

‡ Hazards adjusted for age, sex, SBP, antihypertensive medications, current smoking, prevalent CHD, and body mass index in the groups compared with referent.

HF = heart failure; CHD = coronary heart disease; CVD = cardiovascular disease.
