# Supplementary material for: Prognosis of “pre-heart failure” clinical phenotypes
Source: PLoS One. 2020 Apr 10;15(4):e0231254. doi: 10.1371/journal.pone.0231254 (PMC7147998; doi:10.1371/journal.pone.0231254)
Supplement: S1 Appendix — (DOCX) [file pone.0231254.s006.docx]

**Appendix. Framingham Heart Study criteria for HF diagnosis.**

| **Major Criteria** | **Minor Criteria** |
| --- | --- |
| Paroxysmal nocturnal dyspnea or orthopnea | Pleural effusion by X-ray |
| Distended neck veins | Pulmonary vascular engorgement by X-ray |
| Hepatojugular reflux | Heart rate > 120/minute |
| Rales | Hepatomegaly |
| S3 gallop | Ankle edema |
| Treatment induced weight loss >10lbs/5 days | Decrease in vital capacity by 1/3rd |
| Increased venous pressure > 16 cm water | Dyspnea on ordinary exertion |
| Pulmonary edema, visceral congestion or cardiomegaly on autopsy | Night cough |
| Enlarged heart by X-ray |  |
| Acute pulmonary edema on chest X-ray |  |

Heart failure was diagnosed by the presence of 1 major criterion and 2 minor criteria, or 2 major criteria in the absence of an alternative explanation for findings.
