# Supplementary material for: Prognosis of “pre-heart failure” clinical phenotypes
Source: PLoS One. 2020 Apr 10;15(4):e0231254. doi: 10.1371/journal.pone.0231254 (PMC7147998; doi:10.1371/journal.pone.0231254)
Supplement: S1 Data — (DOCX) [file pone.0231254.s007.docx]

**Statistical Methods for evaluating proportionality of hazards over time**

The assumption of proportional hazards was tested for each Cox proportional hazards model by adding interaction terms for probable HF categorization with natural logarithm of time in years, and possible HF categorization with natural logarithm of time in years. Where these interaction terms were significant, they were kept in the model, which allowed hazard ratios to vary with the natural logarithm of time. These time-specific hazard ratios were estimated, such as the following, using the ESTIMATE statement in PROC PHREG: ln(HR, Possible HF vs Control at t=1 year) = β_1_*(Possible HF status) + β_2_*(Possible HF status)*ln(t).

The proportional hazards assumption was violated for definite HF, CHD, and all-cause mortality outcomes when comparing healthy controls with possible HF and probable HF groups when adjusting for age and sex, and when adjusting for age, sex, and additional covariates. (Results of the interaction tests are presented in **Supplementary Table 2** below). In addition, the interaction term for possible HF with time was significant for the “Other CVD” outcome when adjusting for age, sex, and additional covariates. No violations of the proportional hazards assumption were observed when comparing possible HF and probable HF with each other or with the definite HF group.

Hazard ratios after 1 and 5 years of follow-up estimated from these models with time-varying hazards are presented in **Supplementary Table 3** for models adjusted for age and sex, and in **Supplementary Table 4** for multivariable adjusted models.

**Interpretation**

Probable and possible HF are very high-risk conditions relative to age- sex-matched controls in terms of transitioning to definitive HF; this is understandable as they have partial criteria for HF and need to accrue one or more additional criteria to meet the definition of definite HF, whereas controls do not have such criteria by definition. The very high risk of HF associated with these two pre-HF conditions is particularly evident in the short-run, i.e., during the first year of follow-up where hazards ratios are strikingly high. With the passage of time and the likely depletion of highest-risk individuals with pre-HF (because they transition to definite HF), the relative hazard for HF among the remaining individuals with prevalent pre-HF continues to remain high relative to controls but the magnitude of risk attenuates, more so for possible HF as compared to probable HF.
